# Water temperature and biological sex influence cold pressor pain in healthy adults: a randomized within-subjects trial

**DOI:** 10.3389/fphys.2025.1628111

**Published:** 2025-07-16

**Authors:** Andreas Goreis, Selina Fanninger, Annika Lozar, Anna Mayer, Nina Pfatrisch, Martin Voracek, Paul L. Plener, Oswald D. Kothgassner

**Affiliations:** ^1^ Department of Child and Adolescent Psychiatry, Medical University of Vienna, Vienna, Austria; ^2^ Comprehensive Center for Pediatrics (CCP), Medical University of Vienna, Vienna, Austria; ^3^ Department of Cognition, Emotion, and Methods in Psychology, Faculty of Psychology, University of Vienna, Vienna, Austria; ^4^ University Research Platform “The Stress of Life (SOLE) – Processes and Mechanisms underlying Everyday Life Stress”, University of Vienna, Vienna, Austria; ^5^ Department of Child and Adolescent Psychiatry and Psychotherapy, University of Ulm, Ulm, Germany

**Keywords:** cold pressor test (CPT), water temperature, pain, physiological stress response, within-subjects randomized trial, sex differences

## Abstract

The Cold Pressor Test (CPT) is an established method for evaluating pain perception and stress responses; evidence indicates that females perceive the CPT as more painful than males. However, methodological variations—particularly in water temperature—complicate cross-study comparisons and hinder robust study designs. To address these issues, we examined the effects of three water temperatures (1°C, 3°C, and 6°C) on pain outcomes and physiological stress markers (heart rate [HR] and heart rate variability [HRV]) in healthy adults while exploring sex differences. In a randomized, single-blind, within-subjects trial with 148 participants (68% female), the CPT was administered using a temperature-controlled cooling device with continuous circulation. Participants immersed their dominant hand for up to 3 min, when the trial was terminated. Pain threshold, tolerance, and intensity were recorded alongside HR and HRV. Results revealed significant variations in pain threshold, tolerance, and intensity across temperatures, with lower temperatures eliciting increased pain perception (medium effect sizes). Males demonstrated higher pain tolerance in 1°C and 3°C conditions, with 50% reaching the 3-min cutoff, compared to 39% at 6°C, 23% at 3°C, and 19% at 1°C for females. No significant sex differences were observed for pain intensity, and HR and HRV did not vary across temperatures or between sexes. However, pain was associated with HR and HRV only in males. Our findings underscore the need for meticulous CPT protocol design. Controlling water temperature and implementing appropriate stopping rules—potentially extending beyond 3 min—are critical for improving comparability, replicability, and understanding of pain mechanisms in healthy populations overall.

## 1 Introduction

The Cold Pressor Test (CPT), originally developed by Hines and Brown (1936) for cardiovascular and autonomic nervous system research, has become a pivotal tool in studying human pain and stress responses as well. Typically, the CPT involves the immersion of a limb, usually the hand, into ice-cold water, triggering multiple physiological processes. Pain induction during the CPT is mediated by the activation of cold nociceptors and cold-induced vasospasms, which transmit signals via A-δ and C fibers to the somatosensory cortex (Chen et al., 1989; Klement and Arndt, 1992). This method induces a progressive increase in cold pain and autonomic nervous system (ANS) activity, evidenced by elevated heart rate (HR; Skoluda et al., 2015), blood pressure, and respiratory rate (e.g., Tipton, 1989), alongside a reduction in heart rate variability (HRV; Peng et al., 2015).

The multifaceted stimulation of the CPT renders it a powerful yet intricate experimental method. It is important to note that the specific design of CPT studies depends on their aims: historically, the CPT was used primarily as a tool to induce cardiovascular challenges, which was its original purpose (Hines and Brown, 1936), but it has since been employed extensively as a method to study pain-induced stress responses, particularly in fields such as physiological and biological psychology (Lovallo, 1975; McRae et al., 2006; Skoluda et al., 2015). Its (theoretical) ease of use, safety, and relative consistency of its effects have established the CPT as a crucial experimental method for not only adults but also for children and adolescents (Birnie et al., 2016). Although Hines and Brown (1936) originally framed the CPT as a predictor of hypertension risk, its clinical use is now, among others, as an experimental benchmark for testing analgesics—both opioid (Watso et al., 2022) and non-opioid (Yuan et al., 1998)—and for probing autonomic dysfunction in conditions such as fibromyalgia, anxiety, and posttraumatic stress disorder (Yoo et al., 2020).

Recently, Fanninger et al. (2023) conducted a comprehensive review of over 300 CPT research reports. Two-thirds of these involved healthy adult samples, indicating that most of the literature focuses on healthy adults. This review highlighted significant variability in CPT protocols across studies, which affects outcomes and undermines cross-study comparability. Notably, the review identified a broad range of water temperatures (from −2°C to 12°C, with most studies selecting 1°C, 2°C, or 4°C) and found that more than half of existing CPT studies did not maintain a constant water temperature through circulation, thereby introducing additional confounding factors. To address the impact of water temperature on pain and physiological stress outcomes and to account for individual variability, our study employed, for the first time, a methodical approach to systematically compare pain perception, HR, and HRV in 148 healthy adults undergoing the CPT at controlled temperatures of 1°C, 3°C, and 6°C in a randomized trial with high statistical power. By examining multiple temperatures and carefully controlling experimental conditions, we aimed to enhance the precision of CPT protocols in adults, thereby contributing to the standardization and validity of stress and pain research.

In CPT studies, the primary outcomes include pain threshold (the point at which pain first becomes noticeable), pain tolerance (the duration until the participant withdraws their hand due to unbearable pain), and pain intensity ratings (self-report measures of pain severity during immersion and/or after withdrawal). Often, cutoff times—typically 3 or 4 minutes—are used in CPTs to prevent potential nerve damage from vasoconstrictive tissue hypoxia in highly tolerant participants, which may distort tolerance data and lead to a bimodal distribution of responses, especially at higher temperatures (Chen et al., 1989). For instance, Mitchell et al. (2004) examined 26 participants across four temperatures (1°C, 3°C, 5°C, 7°C) and noted that lower temperatures generally reduced tolerance and increased pain intensity. Despite high test-retest stability, as observed in studies investigating 4°C and 6°C cold water, another study found no notable differences between temperatures in terms of tolerance or intensity (Koenig et al., 2014). Further pertinent research into pain intensity and tolerance across various temperatures includes studies by Hilgard (1969) at 0°C, 5°C, 10°C, and 15°C; Hirsch and Liebert (1998) at 3°C, 8°C, 13°C, 18°C, and 23°C; and Barati et al. (2017) at 1°C, 5°C, 10°C, and 15°C. However, these studies did not primarily focus on the impact of water temperature, resulting in heterogeneous outcomes and diminished analytical power to detect significant effects. This underscores the need for more precise studies to standardize CPT protocols and enhance the utility of the CPT paradigm in pain research.

Sex differences are another source of variation in pain perception, generally and specifically within CPT paradigms. Spanning multiple pain stimuli (Riley et al., 1999), including cold pressor pain (Diotaiuti et al., 2022; Lowery et al., 2003), abundant evidence suggests that women have lower pain thresholds and pain tolerance (Racine et al., 2012) and report higher pain intensity. While motivational (Diotaiuti et al., 2022; see also the seminal work by Obrist, 1976), psychological (Fillingim, 2017), and hormonal (Iacovides et al., 2015) factors play significant roles in the gender-pain differences association, a review of gender-specific CPT studies concluded that sex differences are relatively consistent, suggesting that cold pain sensitivity is more pronounced in women (Fillingim et al., 2009). Moreover, many chronic pain conditions are also more prevalent among women than men (Fillingim et al., 2009; Tan et al., 2020), while the magnitude of autonomic responses—specifically HR and HRV—to pain is generally lower in women (Christou et al., 2005; Reulecke et al., 2016). This observation has prompted some (Kakon et al., 2021; Lentini et al., 2021; Peckerman et al., 1991) to investigate whether pain intensity during the CPT is modulated by autonomic responses—that is, whether HR or HRV correlates with perceived pain. However, to date only one study (Bock et al., 2025) has explicitly tested sex differences in this association, finding that males—but not females—exhibit a positive correlation between autonomic responses and pain intensity in 4°C water. This finding warrants replication.

Taken together, past research on the CPT is characterized by various methodologies aimed at answering various research questions. Despite the widespread use of the CPT paradigm, it evidently lacks sufficient standardization (although there have been proposed standard protocols, see Modir and Wallace, 2010 for adults; Von Baeyer et al., 2005, for children), limiting cross-study comparability. Several methodological aspects—including water temperature, laterality of the immersed hand, and participant sex—vary widely across studies, potentially affecting the precision of the paradigm and the validity of key outcomes.

We have recently reviewed the field of CPT studies on a large scale (Fanninger et al., 2023) and, among other things, have also called for a more thorough approach when conducting the CPT, especially with regard to the use of consistent water temperatures to ensure comparability, replicability, and generalizability. Here, we build on those findings not only to clarify these issues but also to guide the future development of CPT study designs by investigating the effects of three water temperatures (1°C, 3°C, 6°C) on pain outcomes (pain threshold, pain tolerance, and pain intensity) and physiological stress markers (HR and HRV) in healthy adults. We also aim to compare these outcomes between biological sexes to delineate known sex effects in pain induction. Specifically, our hypotheses were:

### 1.1 Confirmatory hypotheses for water temperature

1) Colder water leads to a lower pain threshold than warmer water temperature.
2) Colder water leads to a lower pain tolerance than warmer water temperature.
3) Colder water is associated with higher pain intensity (as measured by the visual analog scale and the McGill Pain Questionnaire) than warmer water temperature.
4) Colder water is associated with a higher HR (beats per minute, bpm) than warmer water temperature.
5) Colder water leads to lower HRV (quantified via the root mean square of successive differences, RMSSD) than warmer water.
6) Colder water is associated with a stronger positive association between HR changes and pain intensity compared to warmer water.
7) Colder water is associated with a stronger negative association between HRV changes and pain intensity compared to warmer water.

### 1.2 Confirmatory hypotheses for sex differences

8) Women have a lower pain threshold than men.
9) Women have a lower pain tolerance than men.
10) Women report higher pain intensity than men.
11) Women exhibit a higher HR than men.
12) Women exhibit lower HRV than men.
13) The positive association between HR changes and pain intensity is more pronounced in men than in women.
14) The negative association between HRV changes and pain intensity is more pronounced in men than in women.

### 1.3 Exploratory hypotheses for menstrual cycle phase and hormonal contraception

Finally, we posited the following exploratory hypothesis:

15) Menstrual cycle phase (follicular vs luteal), hormonal contraception use, and sex are associated with differences in pain threshold, pain tolerance, heart rate, and heart rate variability.

## 2 Methods

This study was conducted in accordance with the Declaration of Helsinki. The study was approved by the Ethics Committee of the Medical University of Vienna (#1434/2021). The study’s preregistration, open data, as well as open code, are accessible at a public repository of the Open Science Framework (https://osf.io/ws37b/).

### 2.1 Procedure

We recruited healthy participants (*N* = 148) aged 18 to 35 from October 2022 to April 2024 through online and offline advertisements, targeting both students (who were offered course credit) and staff at the Medical University of Vienna and the University of Vienna. Eligibility criteria required participants to be in good health and free from any mental disorders or physical illnesses. Exclusion criteria were set to omit individuals with a history of fibromyalgia, rheumatic disease, heart disease, high blood pressure, Raynaud’s disease, frostbite on the arms or legs, chronic pain, or serious injuries to the dominant hand/arm, due to contraindications with the CPT. Additionally, participants who had consumed analgesics, alcohol, or illicit drugs within 24 h prior to the experiment were excluded, as these substances could significantly alter pain sensitivity (al’Absi et al., 2013; Horn-Hofmann et al., 2015). No monetary compensation was offered for participation.

After arriving for their appointment, participants provided written informed consent. Subsequently, we attached an electrophysiological sensor to measure HR and HRV (see Instruments subsection for details). This was followed by a 10-min resting period, during which participants were instructed to sit upright in a chair and relax. Room temperature was always kept constant at 21°C. Prior to each of the three CPT trials, participants neutralized their dominant hand under a stream of running lukewarm water (∼35°C) from a standard tap for precisely 2 min. A researcher supervised this process in a bathroom adjacent to the lab room to ensure compliance and proper hand immersion. After neutralizing their hand and a 3-min rest in the lab room, sitting upright again, instructions for the CPT were given. These preparatory measures were taken to minimize potential confounding variables before the actual water immersion.

Before each of the three CPT trials, participants were instructed to immerse their dominant hand up to the wrist in a container (i.e., the CPT) filled with cold water, following a prompt issued by the researcher. Concurrently with the submersion, the researcher initiated a stopwatch. The hand was to remain submerged in the water until the participant found the pain unbearable (we, therefore, employed a tolerance paradigm). To minimize the risk of adverse events, such as potential nerve damage from vasoconstrictive tissue hypoxia, and to ensure comparability with other studies, the maximum immersion time was capped at 180 s, which is standard in most CPT tolerance paradigm studies (Fanninger et al., 2023). Should this duration be reached, the researcher was to intervene and verbally halt the immersion. Participants were initially unaware of the stopping rule; however, reaching the maximum immersion time of 180 s during any trial revealed that a time limit was in place. Further, participants were also asked to verbally inform the researcher when they first perceived the water as painful, which served as a measure of pain threshold. All participants received the same scripted instructions—read aloud according to our protocol—both for the general CPT procedure and for signaling their pain threshold.

**FIGURE 1:**
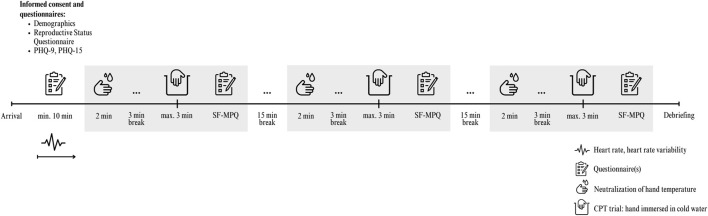
Study overview and timeline of procedures. Note. PHQ, Patient Health Questionnaire; SF-MPQ, Short Form of the McGill Pain Questionnaire.

Throughout the immersion process, the researcher remained in the same room but refrained from conversing or making eye contact with participants. If they withdrew their hand from the water, the stopwatch was stopped; the duration—indicating pain tolerance—was recorded, and participants were then allowed to dry their hand. Subsequently, participants assessed the pain intensity of each trial using the short-form McGill Pain Questionnaire (for intensity during immersion) and a visual analog scale (VAS) for intensity after hand withdrawal (see Instruments subsection). Following the evaluation of pain intensity, a 15-min rest period was observed, during which participants remained seated upright in their chairs. After this interval, the next trial commenced (see Figure 1 for an overview of the study procedure). This procedure was consistently adhered to throughout the study. Participants were blinded to the water temperature, which was randomized and counterbalanced across participants using a computer-generated sequence generator, resulting in six unique temperature sequences.

### 2.2 Instruments

#### 2.2.1 Pain intensity

We assessed pain intensity in response to each of the three CPT trials using two distinct measures to capture both the experienced pain during immersion and the perceived pain immediately after withdrawal.

Participants were presented with the short form of the McGill Pain Questionnaire (SF-MPQ; Melzack, 1987) in German immediately following each CPT trial. The SF-MPQ is designed to evaluate the quality and intensity of pain experienced during the immersion through 15 descriptive items—11 sensory and 4 affective—each rated on a 4-point Likert scale ranging from 0 (“none”) to 3 (“severe”). A cumulative pain intensity score was derived by summing all descriptive item ratings, providing a comprehensive assessment of the pain experienced while their hand was submerged.

Additionally, we also included a Visual Analog Scale (VAS), which was printed on the same sheet of paper. The VAS specifically measured the current pain intensity immediately after hand withdrawal. Participants marked their pain perception along a 100 mm line, anchored at 0 = “not painful at all” and 100 = “worst pain imaginable”, using a pen. This measure captures the immediate aftermath of the CPT, reflecting how participants felt upon removing their hand from the cold water. The reliability (McDonald’s ω) for the three VAS assessments was ω = 0.91. Both the SF-MPQ cumulative pain score (i.e., pain during immersion) and the VAS (i.e., pain after withdrawal) were administered immediately following each CPT trial. Participants’ hands were dried with paper towels while they remained seated, and the questionnaires—placed on an adjacent table—were initiated within seconds of hand withdrawal to ensure accurate, timely reporting of their pain experiences.

#### 2.2.2 Depressive and somatoform symptoms

To ascertain the health status of participants, we administered the depression (PHQ-9) and somatoform (PHQ-15) modules of the Patient Health Questionnaire (Löwe et al., 2002). Both are widely used instruments for the assessment and screening of depressive and somatoform symptoms, respectively. The PHQ-9 assesses the severity of depressive symptoms in the last 2 weeks using nine items, whereas the PHQ-15 assesses how bothered respondents have felt by fifteen common physical complaints in the last 4 weeks. Both instruments were administered during the resting period before the CPT trials began. The reliability was McDonald’s ω = 0.74 for the PHQ-9 and ω = 0.78 for the PHQ-15.

#### 2.2.3 Cold pressor test apparatus

We used a 15 × 22 × 19.5 cm (5.9 × 8.7 × 7.6 inches) refrigerated cooling bath with a capacity of 5.45 L (Julabo-Corio-CD-601F, Julabo GmbH, Seelbach, Germany). The water was maintained at the specified temperature (1°, 3°, or 6°C) with an accuracy of ±0.03°C maintained by a built-in thermostat while circulating continuously at a rate of 15 L per minute. See Supplementary Figure S1 for a photograph of the cold pressor apparatus setup used in the current study.

#### 2.2.4 Physiological measures

We continuously monitored heart rate (HR) in beats per minute (bpm) and heart rate variability (HRV), operationalized as the root mean square of successive differences (RMSSD), throughout the study duration using the movisens EcgMove 4® sensor (movisens GmbH, Karlsruhe, Germany), which operates at a 1024 Hz sampling rate. The sensor, attached to two single-use electrodes from Ambu A/S (Copenhagen, Denmark), was positioned beneath the sternum on the left side. The DataAnalyzer® software (movisens GmbH, Karlsruhe, Germany) facilitated the conversion of ECG signals into time-series data for HR (bpm) and RMSSD (ms), while also automatically detecting and removing artifacts.

RMSSD was selected as the primary HRV index due to its robust sensitivity to parasympathetic (vagal) activity, making it a reliable indicator of short-term autonomic nervous system (ANS) function (Malik et al., 1996). Among the various HRV measures, RMSSD is particularly favored in studies involving acute stressors, such as the CPT, because it effectively captures the rapid, beat-to-beat fluctuations associated with parasympathetic modulation (Billman, 2011) and enables analyses of HRV over short time periods. Unlike frequency-domain measures, which can be influenced by breathing patterns and other artifacts, RMSSD provides a straightforward, comparable, time-domain assessment that is less susceptible to such confounding factors, thereby ensuring greater accuracy and reproducibility (Billman, 2011). We analyzed 3-min segments before each CPT trial to obtain baseline HR/HRV values and used variable-duration segments corresponding exactly to the hand immersion period for HR and RMSSD analysis. Trials with hand immersion durations below 30 s were discarded, resulting in the exclusion of 41 HR trials and 170 HRV trials.

#### 2.2.5 Menstrual cycle and hormonal contraception

The methods used to determine menstrual phase and hormonal contraception status are detailed in the Supplementary Material.

### 2.3 Analysis

The software G*Power (Faul et al., 2007) was used to conduct an *a priori* power analysis aimed at achieving a power of 0.80 to detect a small within-factor effect (for Hypotheses 1–7) and an interaction effect (for Hypotheses 8–14) with an effect size of *f* = 0.10, at the standard alpha error probability of 0.05. According to this power analysis, a minimum sample size of *N* = 130 would be necessary.

All analyses were conducted using R version 4.1.3. For the evaluation of our hypotheses, we employed linear mixed-effects modeling with the R package lme4 (Bates et al., 2015), given that the repeated outcomes from the three CPT trials (i.e., pain threshold, pain tolerance, intensity, HR, HRV) were nested within participants. To address potential skewness in the HRV data (RMSSD values), we applied a natural logarithmic transformation prior to analysis. To address Hypotheses one to seven, the analysis included the fixed factor of temperature (at 1°C, 3°C, and 6°C, factor-coded) with the repeated-measures variables serving as outcomes. For Hypotheses 8–14, the analysis incorporated the fixed factors of temperature (1°C, 3°C, and 6°C, factor-coded) and sex (male and female, factor-coded), alongside their interaction as predictors of the outcomes. Furthermore, to ensure the integrity of our randomized within-subjects design, we conducted a randomization check. We assessed the main effects of sequence order (fixed effects) on each outcome and the interaction between sequence order and sex. Non-significant main effects and interactions indicate that randomization effectively balanced the order in which participants experienced the different CPT temperatures, confirming successful randomization. Wherever significant interactions were identified, simple effect analyses were carried out, and for multiple comparisons, Tukey’s tests were utilized to adjust for the risk of type-1 errors.

## 3 Results

The final sample for analysis comprised 148 participants (mean age = 23.9 years, SD = 3.3). Sex distribution was 68% female and 32% male, with no significant age differences between sexes (p = 0.13). In terms of gender identity, 64% identified as female, 31% as male, 2% as non-binary, and 1% as gender-fluid (see Table 1). Among biological females, 62 exhibited regular menstrual cycles, while 34 used hormonal contraception (oral contraceptives or hormonal intrauterine devices) at testing. Most participants had completed secondary education (71%) or held a university degree (27%). Regarding relationship status, 39% were single and 57% were in a relationship, and 88% reported not smoking regularly. On the PHQ-9, participants’ scores were significantly below the cutoffs for major or mild depressive disorders (Levis et al., 2019), and on the PHQ-15, the sample exhibited very mild somatoform symptoms (M = 5.62, SD = 4.01), slightly above the established cutoff (>5) (Kroenke et al., 2010).

**TABLE 1:** Sample descriptive statistics.

Variable	*n* (%)
Sex	
Female	100 (68%)
Male	48 (32%)
Gender	
Female	95 (64%)
Male	46 (32%)
Non-Binary	3 (2%)
Gender-Fluid	1 (1%)
Highest level of education	
Middle school	1 (1%)
Apprenticeship	1 (1%)
Secondary school	106 (72%)
University degree	40 (27%)
Relationship status	
Single	57 (39%)
In a relationship	85 (57%)
Engaged	3 (2%)
Married	3 (2%)
Smoking cigarettes regularly	
Yes	18 (12%)
No	130 (88%)
Drinking alcohol occasionally	
Yes	128 (91%)
No	13 (9%)
Mental/physical health variable	*M (SD)*
Depressive symptoms (PHQ-9)	4.47 (3.31)
Somatoform symptoms (PHQ-15)	5.62 (4.01)

Note. Percentages may not total 100 due to rounding. The gender variable had 3 missing values, the question of whether alcohol is consumed regularly 7 missing values, and the PHQ-9, and PHQ-15, had 4 missing values each.

### 3.1 Water temperature

**FIGURE 2:**
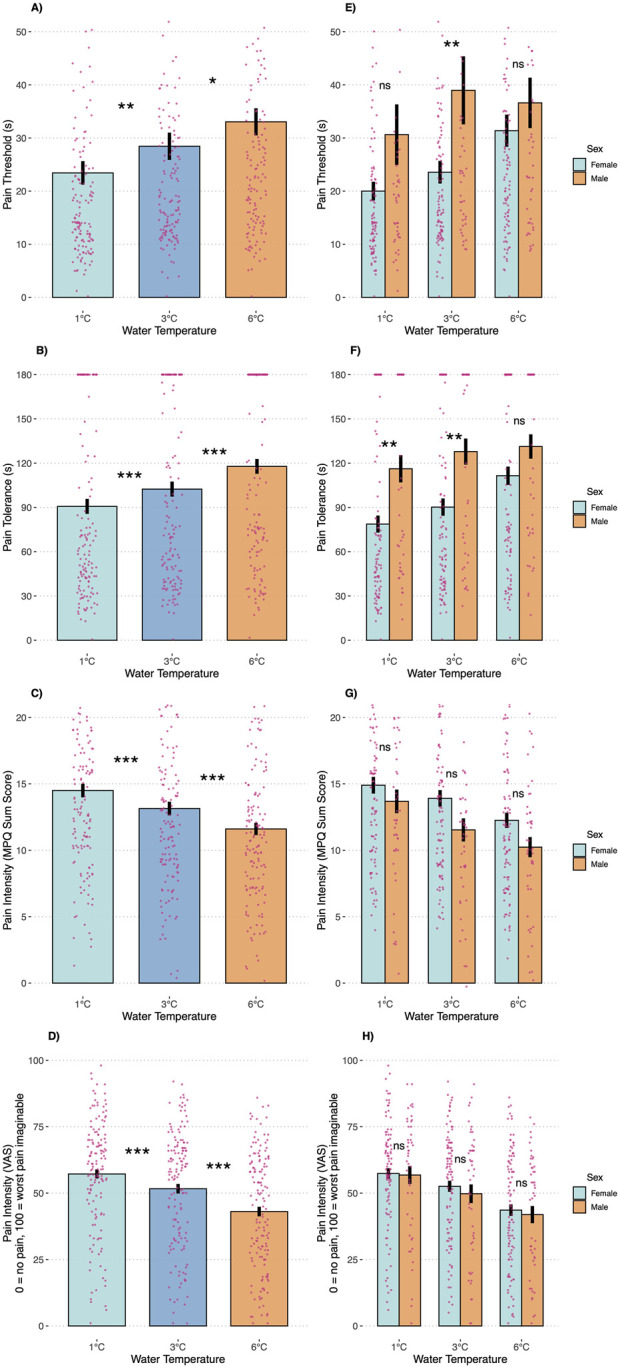
Pain outcomes during the cold pressor test trials by temperature condition **(A–D)** and participant sex **(E–H)**. Note: ns, not significant; *p < 0.05; **p < 0.01; ***p < 0.001. Error bars are SEMs.

**FIGURE 3:**
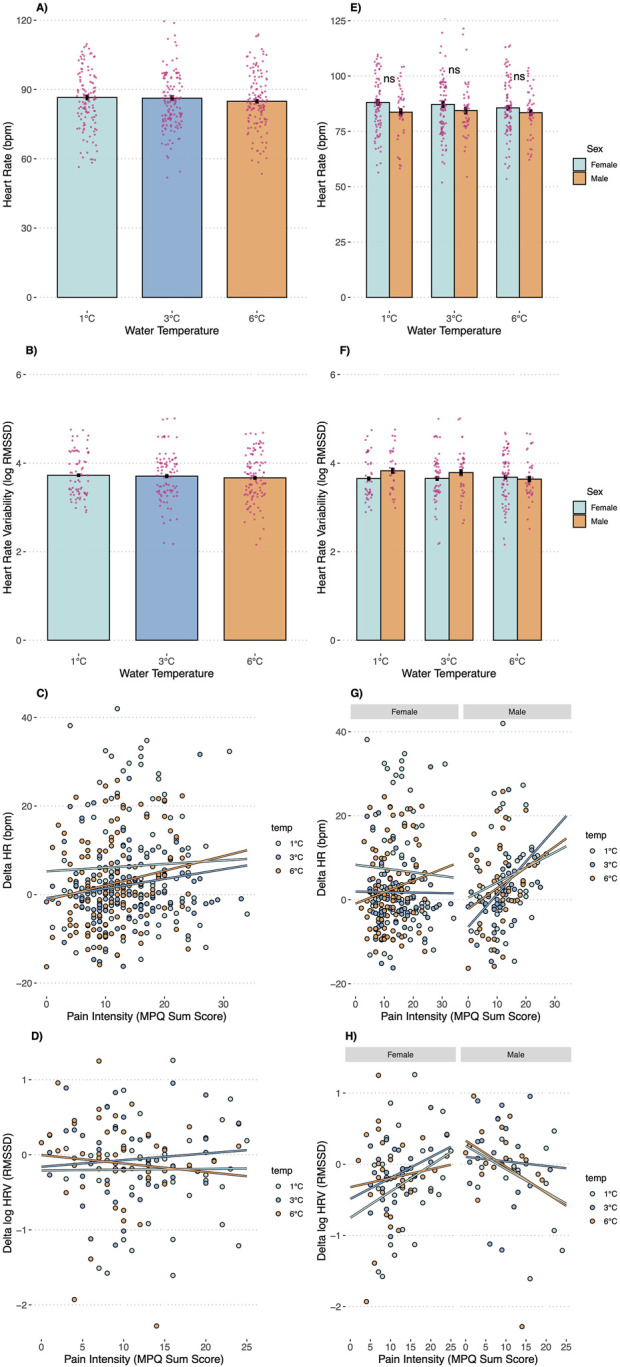
Heart Rate, Heart Rate Variability, the association between changes in Heart Rate (delta) and pain intensity, and the association between changes in Heart Rate Variability (delta) and pain intensity by Temperature Condition **(A–D)** and as a function of temperature and sex **(E–H)**. Note: ns = not significant; * Error bars are SEMs.

The water temperature (1°C, 3°C, or 6°C) significantly affected all pain outcomes (Figures 2A–D) but not physiological stress markers (i.e., HR and HRV; Figures 3A,B). Overall, pain thresholds were lowest at 1°C (M = 23.42 s, SD = 27.00 s), increasing by 5.09 s at 3°C (p = 0.004) and 10.1 s at 6°C (p < 0.001). Pain tolerance was shortest at 1°C (M = 90.69 s, SD = 62.06 s), rising by 11.36 s at 3°C and 27.10 s at 6°C (both ps < 0.001). Notably, 43% of participants reached the 3-min limit at 6°C, compared to 32% at 3°C and 28% at 1°C (see Table 2). Pain intensity during immersion (McGill Pain Questionnaire) and after withdrawal (VAS) followed a similar trend, with 1°C rated significantly more painful than 3°C and 6°C. Specifically, pain at 1°C exceeded that at 3°C with small effect sizes (McGill: d = 0.22; VAS: d = 0.26) and that at 6°C with medium effect sizes (McGill: d = 0.49; VAS: d = 0.66; all ps < 0.001).

**TABLE 2:** Proportion of participants achieving the pain tolerance cutoff of 3 min.

Temperature	% (n) tolerance cutoff (3 min) reached	χ^2^, p
6°C	43% (64)	2.62 (0.106)
Female	39% (39)	
Male	53% (25)	
3°C	32% (47)	10.91 (0.001)
Female	23% (23)	
Male	50% (24)	
1°C	28% (41)	12.29 (<0.001)
Female	19% (19)	
Male	47% (22)	

### 3.2 Sex differences

Our subsequent analyses focused on anticipated sex differences in pain outcomes (Figures 2E–H). A significant temperature-by-sex interaction was observed for pain threshold (F = 3.15, p = 0.045), indicating that men demonstrated a higher pain threshold—15.45 s longer—than women in the 3°C condition (p = 0.046), with no significant differences at 1°C (p = 0.308) or 6°C (p = 0.910). For pain tolerance, men displayed significantly greater tolerance in the 1°C (38.16 s, p = 0.005) and 3°C (37.60 s, p = 0.006) conditions compared to women, whereas no significant difference was observed at 6°C (p = 0.380). Approximately 50% of male participants reached the 3-min stopping rule across conditions, while only 19% of females did so at 1°C (p < 0.001), 23% at 3°C (p = 0.001), and 39% at 6°C (p = 0.106). Pain intensity ratings did not differ significantly between the sexes at any temperature, whether assessed during immersion (McGill: interaction p = 0.144) or after withdrawal (VAS: interaction p = 0.633). Consistent with the temperature-only analyses, no significant sex differences were detected for HR (interaction p = 0.829) or HRV (interaction p = 0.787, Figures 3E,F).

### 3.3 Association between changes in physiology and pain intensity

In the total sample, pain intensity during the CPT was not associated with increases in HR (p = 0.456) or HRV (p = 0.563) at any water temperature (see Figures 3C,D). However, in males, changes in HR were positively associated with pain intensity (b = 0.64, p = 0.032) and changes in HRV were negatively associated with pain intensity (b = −0.04, p = 0.004); these associations were consistent across water temperatures. In contrast, no such associations were observed in females (Figures 3G,H).

### 3.4 Menstrual cycle phases and hormonal contraception (exploratory research question)

Results of our exploratory analyses revealed a significant temperature × menstrual “cycle group” interaction—where cycle group categorizes follicular phase, luteal phase, hormonal contraception users, and men—for pain tolerance (F = 2.80, p = 0.012). At 1 °C, tolerance was lower than in men for the follicular (−45.50 s, p = 0.007) and luteal (−38.08 s, p = 0.030) phases and among hormonal contraceptive users (−36.16 s, p = 0.039). At 3 °C, only contraceptive users differed from men (−46.66 s, p = 0.004), with no significant phase effects (p = 0.057, p = 0.077). No other significant interactions emerged for the remaining outcomes (see Supplementary Material for detailed results).

### 3.5 Randomization checks

We tested the effect of temperature sequence on each outcome and found no significant main effects: pain threshold (p = 0.491), pain tolerance (p = 0.478), pain intensity during immersion (p = 0.433), pain intensity upon withdrawal (p = 0.126), heart rate (HR; p = 0.861), or heart rate variability (HRV; p = 0.374). Additionally, to rule out habituation across repeated exposures, we examined trial-number effects and again observed no significant effects on pain threshold (p = 0.797), tolerance (p = 0.694), intensity during immersion (p = 0.526), intensity upon withdrawal (p = 0.344), HR (p = 0.960), or HRV (p = 0.490). These findings confirm that our randomization successfully balanced temperature order and that neither carryover nor habituation influenced any outcome.

## 4 Discussion

Precision, comparability, and reproducibility are paramount in experimental stress and pain research. This highly-powered, randomized, single-blind, within-subjects trial examined the impact of three water temperatures (1°C, 3°C, and 6°C) on psychophysiological outcomes in 148 healthy adults. We measured pain threshold, pain tolerance, pain intensity, and stress-related outcomes, including HR and HRV. Our results revealed significant differences in pain responses across temperatures and indicated sex-specific physiological patterns: in males, perceived pain was positively associated with HR and negatively with HRV—a relationship not observed in females. Furthermore, our findings corroborate established sex differences, confirming that biologically male individuals exhibit higher pain thresholds and tolerance than their female counterparts.

In CPT trials, selecting an appropriate water temperature is crucial for accurately assessing pain tolerance. Our findings indicate that a substantial proportion of participants reached the 3-min safety cutoff: 43% in 6°C water, 32% in 3°C water, and 28% in 1°C water. This suggests that higher temperatures may not be suitable for investigating individual differences in pain tolerance due to the potential for ceiling effects, particularly among men. Additionally, the commonly used 3-min cutoff—most frequently reported in the literature (Fanninger et al., 2023), with only some studies extending to 4 min or longer—may not be feasible for healthy populations, even at lower temperatures. We hypothesize that these safety cutoffs were originally adopted from clinical research and subsequently applied to healthy participants, a practice that may not be optimal when pain tolerance is the primary research goal, as healthy adults, especially biological males, frequently reach the cutoff. Apart from individuals with Raynaud’s Disease—where vasospasm may persist and critically reduce oxygen supply, and who can be screened and excluded beforehand—higher safety cutoffs, and thus longer exposure to cold water, may be more appropriate for healthy adults. This adjustment could reduce ceiling effects and enhance the sensitivity of pain tolerance measurements in this population.

Regarding pain intensity during immersion and after hand withdrawal, even though the temperature differences were only a few degrees Celsius, the effect sizes for pain intensity differences were in the medium range. However, pain intensity did not differ between sexes at any of the three temperature levels, which is consistent with prior related CPT findings (Stening et al., 2007). However, the duration of hand immersion (i.e., tolerance measures) differs significantly between sexes, with females, on average, terminating the paradigm earlier. Consequently, pain tolerance results may be influenced by these time offsets. When pain intensity is the primary focus of research, such confounding effects can be mitigated by continuously assessing current pain during hand immersion—for example, using a pain slider controlled by participants (Zunhammer et al., 2022) or through continuous verbal assessments—rather than relying solely on single retrospective pain intensity assessments, as was done in our study.

While we observed significant changes in pain threshold, tolerance, and intensity as a function of water temperature, our study did not reveal corresponding differences in physiological stress parameters, suggesting that autonomic measures are not solely determined by water temperature variations. Both HR and HRV—despite exhibiting typical responses during immersion—remained consistent across the three temperature conditions and between sexes. This indicates that pain outcomes may be more sensitive to temperature fluctuations than autonomic responses, which might be influenced by other factors. Moreover, we found that in males, increases in HR and decreases in HRV were associated with higher perceived pain intensity—a pattern consistent with the findings of Bock et al. (2025), which may have implications for the predictive value of the CPT regarding autonomic responses and cardiovascular health. Our design was not optimally configured to capture these associations fully; particularly in the lower temperature trials, many participants (especially females) had immersion times too short for reliable autonomic measurements, necessitating their exclusion from analysis. Overall, our findings suggest that HR and HRV should not be interpreted as standalone objective indicators of pain perception, as has sometimes been implied (Loggia et al., 2011; Tousignant-Laflamme et al., 2005) and critically discussed (Cowen et al., 2015); furthermore, sex must be considered in such analyses. However, we also did not measure blood pressure—a core autonomic variable commonly assessed in CPT research on cardiovascular outcomes (e.g., Lovallo, 1975)—which might have revealed differences not captured by HR and HRV alone (see also Bock et al., 2025, for a comprehensive discussion of sex differences in cardiovascular risk).

Regarding the stress-focused area of CPT literature, some studies have shifted their focus into the direction of more stress-eliciting, socially evaluated, CPT paradigms (Schwabe et al., 2008), wherein physiological pain is augmented by a social-evaluative component, often through videotaping and specific instructions. When the primary interest of CPT-related studies is in physiological or endocrine stress markers (and/or in sex differences), opting for these more stress-eliciting socially evaluated CPT paradigms might be more appropriate than the traditional CPT. Although calls for standardization of protocols for socially evaluated CPTs have been made (Schwabe and Schächinger, 2018), variables such as the precise water temperature may not influence outcomes as significantly as in studies focusing on pain tolerance. Furthermore, our exploratory examination of menstrual cycle phase and hormonal contraceptive use revealed that, compared to men, women in both cycle phases and contraceptive users showed reduced tolerance at 1 °C—and only contraceptive users at 3 °C—but given the modest magnitude and inconsistency of these effects, they should be viewed as hypothesis-generating and warrant targeted follow-up.

On another note, our study offers only limited insight into the conditioned pain modulation (CPM; also known as diffuse noxious inhibitory control) literature—where cold-water immersion serves as the conditioning stimulus followed by a heterotopic noxious test stimulus to probe endogenous inhibitory pathways—a purpose distinct from pain/stress induction research. Although water temperature modulates CPM (Granot et al., 2008, found greater pain inhibition in males than females), our randomized, same-site CPT protocol could only invoke peripheral desensitization or habituation rather than central inhibition. Readers interested in CPM paradigms are referred to comprehensive reviews (e.g., Kennedy et al., 2016).

Our study, while offering a highly-powered examination of water temperature and sex differences in the CPT using state-of-the-art apparatus for valid temperature control, is subject to several limitations. First, although participants were blinded to the water temperature, the research personnel conducting the CPT were not, as they were required to adjust the water temperature. Second, outdoor temperature was not recorded despite our efforts to maintain consistent external thermal conditions (e.g., by controlling room temperature and standardizing hand temperature neutralization before each trial). This factor could have influenced our results (Farbu et al., 2022), considering that data collection took more than 1 year. Furthermore, another limitation of this study pertains to the statistical modeling of CPT tolerance time. Although we utilized linear mixed-effects models, which are known for their robustness to moderate deviations from normality, CPT tolerance time is inherently a strongly right-censored measure. This right-censoring may violate the assumption of normally distributed residuals, potentially impacting the precision of our estimates. Moreover, the exclusion of 41 CPT trials and 170 HRV segments under 30 s—likely reflecting the quickest withdrawals and highest pain responses—could bias our findings by underrepresenting those extreme responders. Furthermore, while our study primarily focused on the biological sexes and did not address gender identity, it is important to emphasize the need for more gender-inclusive research, especially since the scholarly literature on pain perception in transgender individuals is notably sparse (however, see Anger et al., 2024, for a first review). Transgender people frequently face discrimination, which can lead to psychobiological repercussions and various psychopathologies (see, e.g., the minority stress model, Feinstein, 2020). Additionally, other psychopathological conditions, such as non-suicidal self-injury (Koenig et al., 2016), borderline personality disorder (Fales et al., 2021), post-traumatic stress disorder (Tesarz et al., 2020), and chronic pain disorders (Potvin and Marchand, 2016) often exhibit altered pain thresholds or dysregulated pain perception. Studies of these conditions could significantly benefit from the precise and refined CPT methodologies outlined here, as well as from a broader standardization of cold pressor pain paradigms. By understanding the nuances of pain perception and tolerance across different conditions, the field of clinical pain research may gain deeper insights into specific pathologies and the underlying mechanisms that directly influence increased or reduced pain sensitivity.

In conclusion, this study’s key findings underscore the critical role of experimental design choices in ensuring precision and comparability, particularly in the assessment of pain tolerance and intensity. Our principal finding is that, in healthy adults, water temperature in the CPT—when accurately controlled—significantly affects outcomes related to tolerance, threshold, and intensity. Furthermore, maintaining a consistent temperature is essential when comparing sexes. Lastly, we support the finding that the association between perceived pain and autonomic responses is modulated by sex, with males exhibiting higher HR and lower HRV as pain increased—a pattern not observed in females. Given the value of the CPT as a widely used and accessible tool in experimental pain research, we advocate for a careful selection and justification of protocols, including temperature settings, apparatus, and cutoff rules, to enhance comparability and validity. Ultimately, the precise and standardized application of the CPT, particularly in populations with psychopathologies that may alter pain perception, is even more crucial as the field strives to gain reliable and applicable insights into experimental pain processes.

## Data Availability

The datasets presented in this study can be found in online repositories. The names of the repository/repositories and accession number(s) can be found below: Open Science Framework (https://osf.io/ws37b/).
