# Correction: Water temperature and biological sex influence cold pressor pain in healthy adults: a randomized within-subjects trial

**DOI:** 10.3389/fphys.2025.1667543

**Published:** 2025-08-06

**Authors:** Andreas Goreis, Selina Fanninger, Annika Lozar, Anna Mayer, Nina Pfatrisch, Martin Voracek, Paul L. Plener, Oswald D. Kothgassner

**Affiliations:** ^1^ Department of Child and Adolescent Psychiatry, Medical University of Vienna, Vienna, Austria; ^2^ Comprehensive Center for Pediatrics (CCP), Medical University of Vienna, Vienna, Austria; ^3^ Department of Cognition, Emotion, and Methods in Psychology, Faculty of Psychology, University of Vienna, Vienna, Austria; ^4^ University Research Platform “The Stress of Life (SOLE) – Processes and Mechanisms underlying Everyday Life Stress”, University of Vienna, Vienna, Austria; ^5^ Department of Child and Adolescent Psychiatry and Psychotherapy, University of Ulm, Ulm, Germany

**Keywords:** cold pressor test (CPT), water temperature, pain, physiological stress response, within-subjects randomized trial, sex differences

In the published article, there was an error in the **Funding** statement. “The author(s) declare that no financial support was received for the research and/or publication of this article.” The correct **Funding** statement appears below.

