# Supplementary material for: Water temperature and biological sex influence cold pressor pain in healthy adults: a randomized within-subjects trial
Source: Front Physiol. 2025 Jul 16;16:1628111. doi: 10.3389/fphys.2025.1628111 (PMC12307380; doi:10.3389/fphys.2025.1628111)
Supplement: Supplementary file 1 [file Supplementaryfile1.docx]

**Supplementary Material: Cold Pressor Pain is Influenced by Water Temperature and Biological Sex: A Randomized Within-Subjects Trial**

Andreas Goreis^1,2^, Selina Fanninger^1^, Annika Lozar^1^, Anna Mayer^1^, Nina Pfatrisch^1^, Martin Voracek^3,4^, Paul L. Plener^1,2,5^, Oswald D. Kothgassner^1,2^

**Exploratory Analyses: Menstrual Cycle and Hormonal Contraception**

**Method**

To explore our hypotheses concerning menstrual cycle phases in female participants, we utilized the day-count method to retrospectively determine the menstrual phase at the time of testing due to individual variability in menstrual cycle lengths. This method is recommended when exact measures of ovulation are unavailable (Schmalenberger et al., 2021). By this approach, menstrual phases were identified by counting backward from the most recent menses onset date (where 0 represents the first day of menses), under the assumption that the luteal phase length is relatively constant at 12–14 days. Hence, by evaluating the date of the last menses onset and the current menstrual cycle day, we classified the days from 15 days before to 1 day before menstrual onset as the luteal phase. Conversely, the days from the first day after menstrual onset up to 15 days before the next menstrual onset were classified as the follicular phase.

Furthermore, we assessed contraception methods through the reproductive status questionnaire designed for menstrual cycle status (Schmalenberger et al., 2021). This self-report form included queries such as “Are you currently using oral contraceptive pills, contraceptive patches, contraceptive implant or injection in your arm, or a contraceptive vaginal ring?” and “Are you using an Intrauterine Device (IUD)?”, with options for “hormonal (e.g., Mirena)” or “non-hormonal IUD (e.g., copper)”. Participants who answered affirmatively to the first question or indicated the use of hormonal IUDs were categorized accordingly (i.e., as users of hormonal contraceptives).

**Analysis**

In examining our exploratory hypotheses related to potential differences between menstrual cycle phases, the fixed factors of temperature (1°C, 3°C, and 6°C, factor-coded) and the “group” variable, which encompassed menstrual cycle phases (luteal vs. follicular), hormonal contraception use, and male sex, were included.

**Results**

Our exploratory analyses examined how pain outcomes varied among individuals based on their menstrual cycle phase, the use of hormonal contraception, and how these groups differed from men. The female subsample included 30 individuals in the follicular phase, 32 in the luteal phase, and 34 using hormonal contraception at the time of testing. Our findings (illustrated in Figure 2M–R) indicated that neither the individual cycle phase nor the use of hormonal contraception had a significant effect on pain threshold (interaction between temperature and “cycle group”, i.e., the variable representing cycle phase/contraception/male sex: *p* = .186). However, when considering pain tolerance, a significant interaction between temperature and group was found (*F* = 2.80, *p* = .012). This interaction revealed that in the 1°C condition, both menstrual cycle phases (follicular: -45.50 s, *p* = .007; luteal: -38.08 s, *p* = .030) and the hormonal contraception group (-36.16 s, *p* = .039) exhibited significantly lower pain tolerance compared to men. In the 3°C condition, only participants using hormonal contraception significantly differed from men, showing a lower tolerance (-46.66 s, *p* = .004), whereas no significant difference was observed between either cycle phase and men (*p* = .057 for follicular and *p* = .077 for luteal, respectively). No differences in pain tolerance were identified in the 6°C condition. For all other measured outcomes, including pain intensity, HR, and HRV, the variable of cycle group did not significantly influence the results (all interaction *p*-values > .209).

**Supplementary Figure 1:** Picture of the Cold Pressor Apparatus setup (Julabo-Corio-CD-601F, Julabo GmbH, Seelbach, Germany) used in the study. A hand-crafted red card (shown in the picture) was attached with adhesive tape to conceal the temperature reading, ensuring that the participants were blinded to the water temperature.
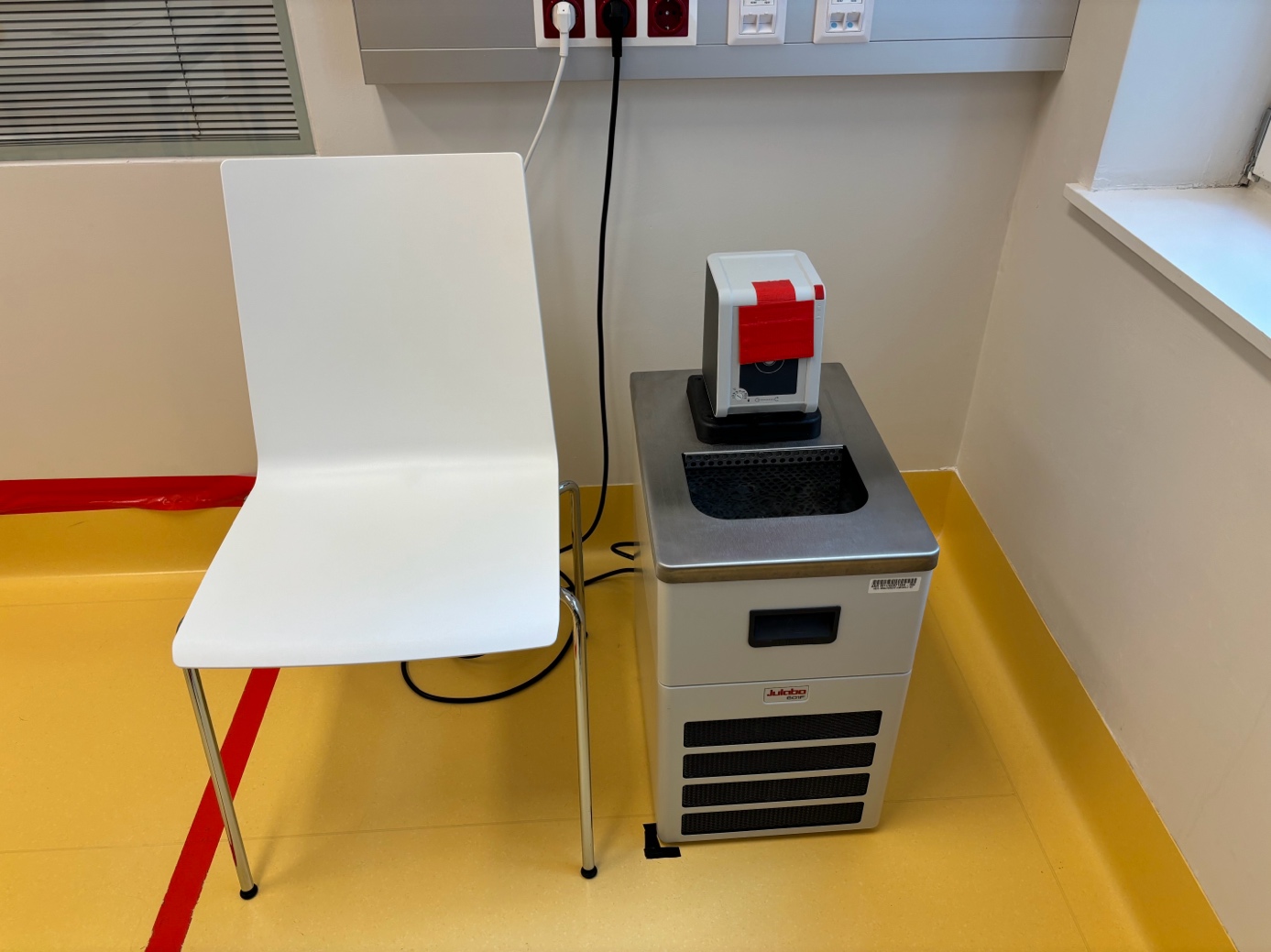


**References**

Schmalenberger, K. M., Tauseef, H. A., Barone, J. C., Owens, S. A., Lieberman, L., Jarczok, M. N., Girdler, S. S., Kiesner, J., Ditzen, B., & Eisenlohr-Moul, T. A. (2021). How to study the menstrual cycle: Practical tools and recommendations. *Psychoneuroendocrinology*, *123*, 104895. https://doi.org/10.1016/j.psyneuen.2020.104895
